# Significance of overexpression of alpha methylacyl-coenzyme A racemase in hepatocellular carcinoma

**DOI:** 10.1186/1756-9966-27-2

**Published:** 2008-05-15

**Authors:** Wei Li, Philip T Cagle, Rafael C Botero, John J Liang, Zhaoping Zhang, Dongfeng Tan

**Affiliations:** 1 Department of Pathology, University of Texas Health Science Center at Houston, Houston, TX 77030, USA; 2 Department of Pathology, Methodist Hospital, Houston, TX 77030, USA; 3 Division of Gastroenterology and Hepatology, University of Texas Health Science Center at Houston, Houston, TX 77030, USA; 4 Department of Pathology, Penn State Milton S. Hershey Medical Center and College of Medicine, Hershey, PA17033, USA; 5 Department of Molecular Genetics, University of Texas, M.D. Anderson Cancer, Houston, TX 77030, USA; 6 Department of Pathology, University of Texas, M.D. Anderson Cancer, Houston, TX 77030, USA

## Abstract

**Background:**

alpha-Methylacyl-CoA racemase (AMACR), an immunomarker for prostatic adenocarcinoma, has been shown to be expressed in a variety of other neoplasms. This study aims to evaluate immunohistochemical expression of the AMACR in neoplastic and nonneoplastic liver lesions, and assess its value in the diagnosis of hepatocellular carcinoma (HCC).

**Methods:**

Formalin-fixed paraffin-embedded tissue sections of 51 HCC (14 well, 22 moderately and 15 poorly differentiated), 9 hepatocellular adenoma (HCA), 48 cirrhotic nodules (CN) and 16 normal liver tissues (NLT) were immunostained for AMACR.

**Results:**

Expression of AMACR is significantly enhanced in HCC tissue compared with non-HCC tissue. High expression of AMACR was found in 82% of HCC including 86% of well-differentiated HCC. In contrast, only 11% of HCA, 13% of CN and 6% of NLT showed high expression for AMACR. Clinicopathological evaluation showed a significant correlation between AMACR expression and venous invasion and capsular invasion by HCC.

**Conclusion:**

Our results suggest that AMACR staining may serve as a useful marker for the differential diagnosis of well-differentiated HCC from HCA. Increased AMACR expression and its association with tumor venous invasion suggest that AMACR may play a role in HCC development and progression.

## Background

Hepatocellular carcinoma (HCC) remains one of the most common malignant neoplasms in the world, with approximately 1 million new cases per year [1]. It is associated with a variety of risk factors, including hepatitis viruses B and C, environmental carcinogens, and genetic disorders. The exact parthenogenesis of HCC remains unclear [2]. The prognosis of HCC is generally poor; therefore, the accurate diagnosis is critical for successful treatment and clinical outcomes [2,3].

Despite many clinical aspects are taken into consideration for the diagnosis of HCC, histological examination still represents the gold standard. However, well differentiated forms of HCC can be difficult to separate histomorphologically from benign lesions such as hepatocellular adenoma (HCA), particularly in small biopsies [4,5]. Because distinction between HCC and HCA is vitally important in determining appropriate therapy and assessing prognosis, many attempts have been made using immunohistochemical stains to aid in such differential diagnosis [6]. However, the clinical application and reproducibility of these immunostains still need to be further investigated [7].

*α*-methylacyl-coenzyme A racemase (AMACR), a peroxymal mitochondrial enzyme involved in the *β*-oxidation of branched-chain fatty acids and fatty acid derivatives, is an enzyme normally present in the peroxisome and mitochondria of renal tubular epithelial cells and hepatocytes. AMACR was initially identified as a molecular marker for prostate cancer on the basis of complementary DNA (cDNA) microarray technology [8-10]. Various degree of expression of AMACR has also been reported in other types of neoplasms including HCC [11-13]. However, the diagnostic value of AMACR staining in HCC, especially in distinction between HCC and HCA, has not been explored. Furthermore, clinicopathological relevance and significance of AMACR expression in HCC remain unknown. In the present study, we evaluated immunohistochemical expression of the AMACR in neoplastic and non-neoplastic liver lesions, and assessed the potential diagnostic utility of AMACR in differential diagnosis of HCC. The correlation of AMACC staining with clinicalpathological factors of HCC was investigated.

## Methods

### Patients and Specimens

The study group was composed of patients submitted to orthotopic liver transplantation or tumor resection at Memorial Hermann Hospitals, affiliated hospital of University of Texas Health Science Center at Houston. Surgical pathology cases were evaluated, including 51 HCC (14 well-, 22 moderately and 15 poorly differentiated HCC) with various pathological features, 9 cases of hepatocellular adenoma and 16 normal liver tissues. Cirrhotic nodules (48 cases) are taken from non-cancerous tissue 1 cm away from the tumor margin. Normal liver tissue excised from traumatically injured liver (8 cases) and near hepatocellular adenoma (8 cases) was reviewed. The HCC were categorized into well (G1), moderately (G2), or poorly (G3) differentiated types, corresponding to Edmondson's grades I/II, III, or IV, respectively [14,15].

The age of patients with HCC ranged from 48 to 73 years, with a mean age of 64.4 years. Of the 51 patients, 42 were men and 9 were women. The etiology of chronic liver disease associated with HCC includes chronic hepatitis C virus (HCV) infection (26 cases), chronic hepatitis B virus (HBV) infection (3 cases), alcoholic cirrhosis (18 cases), primary biliary cirrhosis (1 case), hemochromatosis (1 case) and cryptogenetic cirrhosis (2 cases). Patients with HCA ranged in age from 26 to 48 years (mean, 33 years) with a female-to-male ratio of 6:1. An association with steroid hormone use was not recorded in these cases. Tissues from the specimens were fixed in 10% buffered formalin, processed, and stained with hematoxylin and eosin. All cases were reviewed to confirm the diagnosis independently by 2 pathologists.

### Immunohistochemical Staining

Immunohistochemical stains were performed on formalin-fixed and paraffin-embedded 4-μm sections. The tissue sections were deparaffined, and antigen retrieval conditions included 0.1 M citrate buffer (pH 6.0) in an 800-W microwave oven for 15 minutes. The sections were incubated in 3% hydrogen peroxidase to quench endogenous tissue peroxidase for 5 minutes. The tissue sections were then incubated with a monoclonal antibody against P504S at 1:80 dilution for 30 minutes at room temperature (Zeta Corp., Sierra Madre, CA). The slides were stained in an automated immunostainer using a standard avidin-biotin complex staining procedure. Immunohistochemical reactions were developed with diaminobenzidine as the chromogenic peroxidase substrate, and slides were counterstained with hematoxylin. Prostatic carcinoma served as the positive control. Negative controls were performed for all cases and consisted of identically prepared slides that were treated with antibody diluent (Dako Corp.) in place of primary antibody, but otherwise subjected to the same immunohistochemical staining protocol.

### Assessment of Immunohistochemical Staining

Positive AMACR expression was defined as cytoplasmic staining with either a finely stippled or coarsely granular pattern, which could be easily identified at low-power magnification (<= 100×). Scant faint finely granular background staining, which could not be seen at low-power magnification (<= 100×), was interpreted as negative staining (background staining). Staining intensity was graded as 0 (background staining), 1+ (weak), 2+ (moderate), or 3+ (strong). Staining intensity was further grouped into low staining intensity (grade as 0 or 1+) and high staining intensity (grade as 2+ or 3+) for comparison.

### Statistics

Mean values and standard deviations were calculated to describe the data population. Statistical analyses were performed using the Fisher exact test or two-tailed *t *test. A *P *value of < 0.05 was considered to be significant.

## Results

### Immunohistochemical expression of AMACR in HCC and non-HCC tissue

The distribution of AMACR protein expression in liver tissue was examined by means of immunohistochemical analysis of tissue samples. For statistical purpose, the intensity of AMACR staining was scored using two scales: "high expression" represented moderate (2+) or strong (3+) staining while "low expression" indicated background (0) or weak (1+) staining.

**Figure 1:**
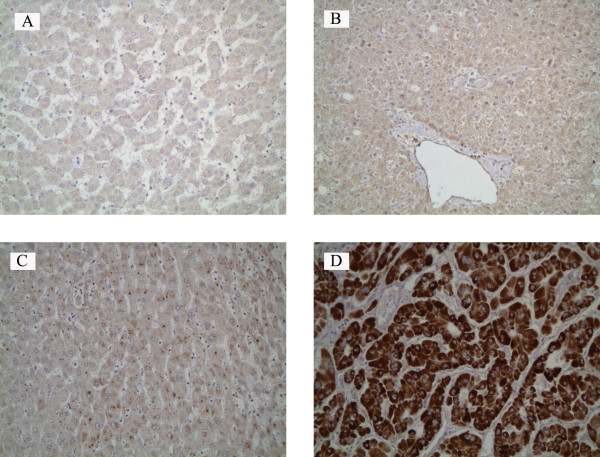
**Expression of AMACR in HCC and non-HCC tissue**. A, normal liver tissue (background staining, grade 0). B, hepatocellular adenoma (background staining, grade 0). C, cirrhotic nodules (weakly positive, grade 1+). D, well-differentiated hepatocellular carcinoma (strongly positive, grade 3+). (immunohistochemical staining; original magnification ×200)

Staining of the AMACR protein was detected in the cytoplasm of both HCC and non-HCC tissue. AMACR staining intensity was generally low (grade 0 or 1+) in NLT, HA, and CN samples (Fig. 1A–C) while the vast majority of HCC samples showed high AMACR expression(grade 2+ or 3+) (Fig. 1D). The results of AMACR immunohistochemical staining for HCC, HA, CN and NLT were summarized in Table 1. The high expression of AMACR was present in 82% (42/51) of HCC. In contrast, only 11% (1/9) of HCA, 13% (6/48) of CN and 6% (1/16) of NLT showed high expression for AMACR. The difference in AMACR high expression between HCC group and non-HCC group was highly statistically significant (<0.001).

**Table 1:** Expression of AMACR in HCC, HCA, CN and NLT

	AMACR staining intensity* n (%)				
		
	Low expression		High expression		
			
	0	1+	2+	3+	Total No. of Cases
HCC	0(0)	9(18)	16(31)	26(51)	51
WDHCC	0(0)	2 (14)	5 (36)	7 (50)	14
MDHCC	0(0)	4 (18)	6 (27)	12 (55)	22
PDHCC	0(0)	3 (20)	5 (33)	7 (47)	15
HCA	6(67)	2 (22)	1 (11)	0 (0)	9
CN	16 (33)	26 (54)	6 (13)	0 (0)	48
NLT	13(81)	2(13)	1(6)	0(0)	16

Note: WDHCC indicates well differentiated HCC;MDHCC: moderately differentiated HCC;PDHCC: poorly differentiated HCC;

HCA: Hepatocellular adenoma; CN: Cirrhotic nodules, NLT: normal liver tissue

*Staining intensity is graded as 0 (scant faint finely granular background staining), 1+ (weak), 2+ (moderate), or 3+ (strong).

Among 51 cases of HCC, high expression of AMACR was found in 86% (12/14) well-differentiated HCC, 82% (18/22) moderately differentiated HCC and 80% (12/15) poorly differentiated HCC. In both low grade (well- and moderately differentiated) and high grade (poorly differentiated) HCC, AMACR high expression was much more frequent compared with HA, CN and NLT (p < 0.001). No significant correlation was found between AMACR expression and HCC grade.

High expression of AMACR was significantly more frequent in CN (13%) than in NLT (6%) (p < 0.01). No difference of AMACR expression was observed between CN and HA, and between HA and NLT. AMACR showed no staining in other types of cells of the liver, including bile duct epithelium, endothelial cells, Kupffer cells, and stromal cells.

### Correlation between AMACR expression and clinicopathological parameters of HCC

Clinicopathological parameters were compared between groups of high and low AMACR expression and the results were listed in Table 2. High-intensity staining appeared to correlate significantly with venous invasion (*P *< 0.05) and capsular invasion (*P *< 0.05). There was no significant correlation between the expression of AMACR and other clinicopathological variables (Table 2).

**Table 2:** Correlation between AMACR expression and clinicopathological features of HCC

		AMACR expression n (%)		
			
Variables	n	Low	High	p-value
Age(yr)				
≥60	28	5(18)	23(82)	NS
<60	23	4(17)	19(83)	
Gender				
male	42	8(19)	34(81)	NS
female	9	2(22)	7(78)	
Tumor size				
≥5 cm	16	3(19)	13(81)	NS
<5 cm	35	8(23)	27(77)	
Histologic grade				
well	14	2(14)	12(86)	NS
mod	22	4(18)	18(82)	
poor	15	3(20)	12(80)	
No. of Tumors				
Solitary	32	7(22)	25(78)	NS
Multiple	19	3(16)	16(84)	
Venous invasion				
Yes	9	0(0)	9(100)	<0.05
No	42	14(33)	28(67)	
Capsular invasion				
Yes	34	1(3)	33(97)	<0.05
No	17	6(36)	11(64)	
Capsular formation				
Yes	38	6(16)	32(84)	NS
No	13	3(13)	10(77)	

## Discussion

AMACR has been established as a valuable diagnostic marker for prostate carcinoma with high sensitivity and specificity [8,9]. Despite many studies on AMACR expression in human cancer [11,16-18], few studies are available describing the role of AMACR expression in HCC. Published reports are limited to the assessments of the frequency and distribution of its expression in HCC and non-HCC tissues [11,13]. Studies by Guzman *et al*. showed various degree and pattern of AMACR expression in HCC and non-HCC tissues. In their study, the cases of hepatocellular adenoma were not included and association between AMACR expression and clinic pathological parameters in HCC was not assessed [13].

In this study, the AMACR protein was detected in the cytoplasm of normal hepatocytes, and the staining were either scant faint (background staining) or weakly positive. These findings are consistent with results previously published [13]. We demonstrated that AMACR expression is significantly increased in the HCC compared with HCA, CN and NLT with respect to the intensity of immunostaining. The high expression of AMACR was found in 82% (42/51) of HCC, while only 11% (1/9) of HCA, 13% (6/48) of CN and 6% (1/16) of NLT showed high expression for AMACR. These findings suggest that AMACR may be involved in the hepatocarcinogenesis. AMACR is important in *β*-oxidation of branched-chain fatty acids. From pathogenesis point of view, there is a possibility that a molecular link exists between this metabolic fatty acid enzyme and HCC. Experimental studies showed that overexpression of acyl-CoA oxidase, regulated by AMACR, can transform cells in vitro [19].

Separation of HCC from HCA, in particular the well-differentiated variant, is a difficult challenge for pathologists. Although helpful morphological criteria have been worked out, there are still cases that cannot be reliably resolved in routine practice [4]. Clinical parameters such as sex, age, history of steroids, hepatitis or level of alpha-fetoprotein may give indications, but not proof, in the differential diagnosis. Since methods of treatment such as embolization and surgery, are different for HCA and HCC, an early correct diagnosis is of major importance. Therefore, it is critical to develop relevant sensitive markers to assist in making an accurate diagnosis. We assessed the potential diagnostic utility of AMACR in HCC. In our study, high expression of AMACR was found in 82% of well-differentiated HCC. In contrast, only 11% of HCA showed high expression for AMACR. These results suggest that AMACR may serve as a useful marker to facilitate accurate diagnosis in specific settings, particularly in distinguishing well-differentiated HCC from HCA given that high AMACR expression is at much lower frequency in HCA. Results may warrant further studies using large volume samples to evaluate the value of AMACR as a diagnostic marker in HCC.

Analysis of AMACR expression in relation to clinicopathological features showed that high AMACR in the HCC was significantly associated with venous invasion. This data suggests an important role of AMACR in tumor invasiveness and progression of HCC. Venous invasion is one of the most important pathological features that lead to postoperative tumor recurrence after resection of HCC [20,21]. The exact mechanism of venous invasion in HCC remains unclear, but active neovascularization of the tumor is likely to play an important role. It is not clear whether and to what extent AMACR is involved in angiogenesis during HCC progression. Further studies are needed to clarify the mechanisms.

Finally, the significance of our findings is that it may have a potential target of therapy in HCC. High expression which is present in a high percentage of HCC, but in only small percentage or rarely in non-HCC tissue, suggests that AMACR may be a target for cancer treatment by using AMACR antibodies or enzyme inhibitors. In addition, individuals with congenital absence of this enzyme have no or only insignificant resultant clinical manifestations [22,23], suggesting that no significant adverse effects will occur in patients treated with anti-AMACR antibody or enzyme inhibitors.

## Conclusion

In summary, our study provides evidence that immunohistochemical detection of AMACR can be helpful in the differential diagnosis between HCC and HCA, especially in tumors of which histology alone is not sufficient for a proper diagnosis. Overexpression of AMACR in HCC and its significant correlation with venous invasion indicate that AMACR may be involved in HCC development and progression. The molecular mechanism(s) of AMACR in HCC merits further investigation.

## Competing interests

The authors declare that they have no competing interests.

## Authors' contributions

WL performed experiments and wrote the manuscript. PTC, ZZ and DT helped perform experiments. RCB, DT and JJL helped write the manuscript.
